# Evidence-based clinical engineering: Health information technology adverse events identification and classification with natural language processing

**DOI:** 10.1016/j.heliyon.2023.e21723

**Published:** 2023-10-31

**Authors:** Alessio Luschi, Paolo Nesi, Ernesto Iadanza

**Affiliations:** a Department of Information Engineering, University of Florence, Via di S. Marta, 3, 50139, Firenze (FI), Italy; b DISIT Lab, University of Florence, Via di S. Marta, 3, 50139, Firenze (FI), Italy; c Department of Medical Biotechnologies, University of Siena, Via A. Moro, 2, 53100, Siena (SI), Italy

**Keywords:** Clinical engineering, Natural language processing, Real-world evidence, Health information technologies, Medical devices

## Abstract

The primary goal of this project is to create a framework to extract Real-World Evidence to support Health Technology Assessment, Health Technology Management, Evidence-Based Maintenance, and Post Market Surveillance (as outlined in the EU Medical Device Regulation 2017/745) of medical devices using Natural Language Processing (NLP) and Artificial Intelligence. An initial literature review on Spontaneous Reporting System databases, Health Information Technologies (HIT) fault classification, and Natural Language Processing has been conducted, from which it clearly emerges that adverse events related to HIT are increasing over time. The proposed framework uses NLP techniques and Explainable Artificial Intelligence models to automatically identify HIT-related adverse event reports. The designed model employs a pre-trained version of ClinicalBERT that has been fine-tuned and tested on 3,075 adverse event reports extracted from the FDA MAUDE database and manually labelled by experts.

## Graphical abstract

**Figure:**
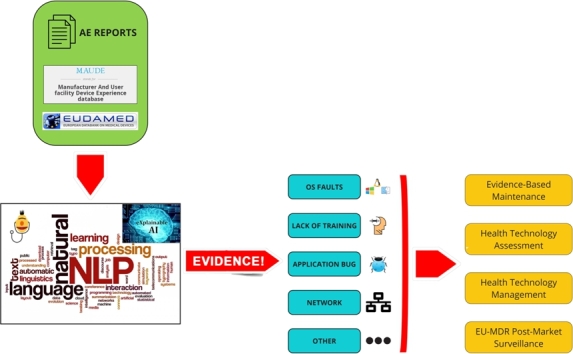

## 1 Introduction

**Figure 1:**
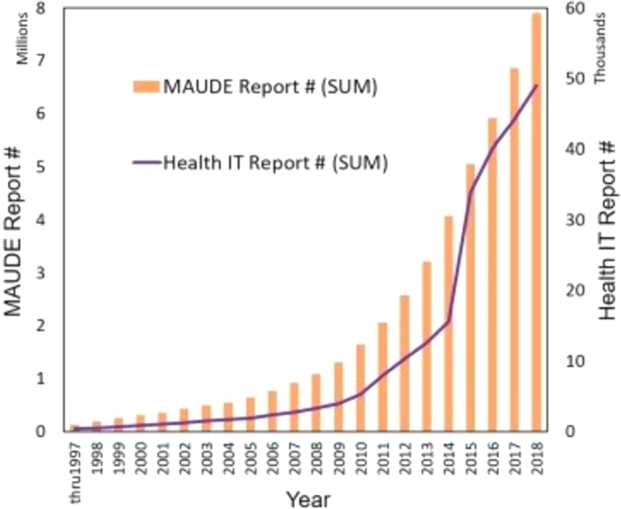
HIT-related adverse events extracted from the MAUDE database up to 2018 [12].

Patient safety is an essential element to guarantee high levels of quality of care [1]. Patient safety is strictly related to health technologies, including devices, medicines, vaccines, procedures, and systems. Studying and managing health technology adverse events is critical for improving medical quality and safety [2]. In recent years, we have encountered a process of critical analysis of the manufacturer's maintenance recommendations, urging Clinical Engineers (CE) and Health Technology Management (HTM) professionals to adopt evidence-based methods to maintain medical equipment's dependability and safety while using their resources wisely [3]. In this scenario, Real-World Data (RWD), i.e., observational data associated with outcomes in real-world settings, can be used to generate Real-World Evidence (RWE) to assess the effectiveness and the safety of a given health technology by examining the intended and unintended consequences of its use. RWE can be employed in healthcare for different purposes, such as to support more effective and cost-efficient medical product regulatory decision-making across the product life cycle. The new EU Medical Device Regulation 2017/745 (EU-MDR) requires companies to register their devices in the EUDAMED database following the European Medical Device Nomenclature (EMDN) [4], and to provide a Periodic Safety Update report and a Post Market Surveillance (PMS) report [5]. The continuous analysis of the safety signals, which emerge from the adverse events of the medical devices available on the market, has indeed a strong significance for manufacturers in relation to the aforementioned legal obligations. Besides, RWE is also very useful for performing the market evaluation of a specific medical device, analysing faults, planning updates and interventions, and avoiding recalls. It is also well-recognized that RWD is a source for assessing the impact of health technologies in terms of risk minimisation, pricing, and reimbursement decisions [6]. Health Technology Assessment (HTA) is “a multidisciplinary process that uses explicit methods to determine the value of health technology at different points in its life-cycle. The purpose is to inform decision-making in order to promote an equitable, efficient, and high-quality health system” [7]. The process is formal, systematic, and transparent, and uses state-of-the-art methods and data collected during the routine delivery of health care to consider the best available evidence [8]. Outcomes can be used to highlight both the most common faults and the unexpected new problems of medical devices. Maintenance is another essential component of the activities in the hospital's CE and HTM departments, because of the enormous personnel and financial resources required. As a result, evaluating the efficiency of maintenance programmes solely depends on making the best use of the available resources [9]. Evidence-Based Maintenance (EBM) involves the use of RWD and scientific RWE to identify the optimal maintenance strategies for medical devices, by analysing the causes of equipment failures to monitor the maintenance effectiveness and plan any necessary changes to improve it [10]. Analyses based on RWE require the availability of a significant amount of RWD to perform a solid study and extract actual evidence of a general nature. Medical RWD can originate from different sources, such as Electronic Health Records (EHR), patient surveys, Computerized Maintenance Management System (CMMS) software [11], and Spontaneous Reporting System (SRS) databases. Recently, these data sources show the common trend of the gradual growth of adverse events related to Health Information Technologies (Fig. 1) [12], which is coherent with the diffusion of medical software in healthcare and with the resulting possible faults which, in addition, may be also caused by the hardware they are installed on [13].

In such a scenario, using Neural Networks and Natural Language Processing (NLP) techniques to mine structured data from the above-cited sources can improve the extraction of RWE, thus empowering the processes of designing, assessing, evaluating, and managing health technologies. In such real-world applications, explainability and transparency of Artificial Intelligence (AI) systems are becoming more and more essential for users and for the researchers and developers who create AI solutions [14]. Explainable Artificial Intelligence (XAI) is a research field that aims to make AI systems results more understandable to humans. The goal of enabling explainability in Machine Learning (ML) “is to ensure that algorithmic decisions, as well as any data driving those decisions, can be explained to end-users and other stakeholders in non-technical terms” [15].

The main goal of this work is to develop a framework to support the extraction of RWE through NLP, Deep Neural Networks (DNNs), and Explainable AI for mining and classifying Health Information Technologies (HIT) adverse events extracted by heterogeneous sources of RWD. To achieve the proposed goal, records extracted by the US Manufacturer and User Device Experience (MAUDE) database have been labelled by experts as HIT/non-HIT adverse events and used to fine-tune a pre-trained model for binary text classification. The model has been validated with a 10-fold validation process and then tested against a subset of records to assess its performance. XAI methods have been further applied to highlight the most common features which led to a given classification, helping the final user understand the type of HIT-related adverse event. The developed framework is something that has not been experienced yet, and so it may result in a novel and possibly new successful approach for obtaining RWE for decision-making purposes in several Clinical Engineering fields, such as Evidence-Based Maintenance, Health Technology Management and Assessment, and Post-Market Surveillance.

## 2 Background

### 2.1 Data sources available for medical device vigilance

Health authorities maintain two types of regulatory databases: SRS databases and recall/alert databases. The main publicly available SRS databases are:

• The US Manufacturer and User Facility Device Experience (MAUDE), regulated by the FDA Center for Devices & Radiological Health;
• The EU European Databank for Medical Devices (EUDAMED), regulated by the European Commission;
• The Australian Database of Adverse Event Notifications (DAEN), regulated by the Therapeutics Goods Administration.

Manufacturing companies are forced to report events to vigilance databases; individuals, healthcare providers, patients, and other organisations are free to report events of their own volition. This voluntary reporting is indeed a significant contributor to the problem of under-reporting. In 2013, the FDA introduced a final ruling requiring the implementation of a Unique Device Identification (UDI) system for medical device vigilance. Similarly, the European Commission mandated UDI adoption for devices certified under the EU-MDR starting in 2020. MAUDE is the most widely used and publicly accessible SRS for adverse events, gathering data from all across the world [16]. EUDAMED is still relatively short in data because the event submissions started in 2022. Moreover, the public database access limits are still unknown.

### 2.2 Spontaneous reporting systems and health information technologies fault classification

A literature review has been performed on SRS databases and their use for data and text mining, as well as on HIT fault classification. The majority of works in the area of mining SRS databases were based on the FAERS and the VAERS but also included articles based on the MAUDE. Very few of them regarded data repositories maintained by other countries. Identified articles focused only on structured data. Lately, several researchers have started tuning the SRS disproportionality findings using data from additional data sources [17], [18], [19]. Alemzadeh et al. [20] examined 5,294 medical device recalls from 2006 to 2011 and found that 1,210 of the recalls during that time were related to computers. 94% of them carried a risk of death or serious injury. Software bugs accounted for 64%.

An analysis of MD recalls registered in FDA records for the period 1999-2005 reported that one-third concerned MDs using software for their functioning and showed a constant increase of software failure throughout these years [21]. Pecoraro and Luzi [22] analysed 3,745 reports related to software failure on HIT extracted from the MAUDE database, classifying them into ten classes.

### 2.3 Natural language processing

**Figure 2:**
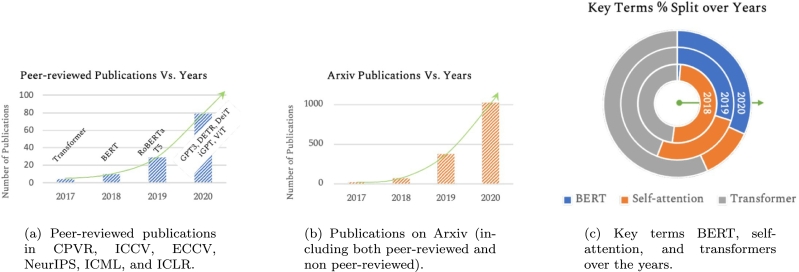
Peer-reviewed and non-peer-reviewed publications for key terms BERT, self-attention, and transformers over the years [31].

Finally, a literature review has been performed on NLP techniques applied to medical devices. Sentiment Analysis (SA) is an approach to NLP that identifies the emotional tone behind a body of text. In healthcare, it can be employed to gain insights from both medical social media and clinical documents regarding the effectiveness of a treatment or medication [23]. A recently published scoping review was used as starting point [24]. The first distinction that emerges from the review is the preferred approach used for NLP tasks and SA: lexicon-based vs ML-based. As generally stated by the authors, the lexicon-based approach is not well suited for capturing the meanings in medical texts. The majority of analysed papers rely on ML methods (Support Vector Machines - SVM, Naïve-Bayes, regression tree) using input features such as POS (Part of Speech) tagging, TD-IDF (Term Frequency-Inverse Document Frequency), BTO (Binary Term Occurrences) and Word2Vec. In particular, the SVM classifier is one of the most successfully used in opinion mining [25], [26]. Biomedical texts, including adverse events reports, are potential resources of massive information and hidden knowledge, unfortunately, it is not possible for researchers and practitioners to keep themselves updated with all the developments in the biomedical field [27]. The emphasis of biomedical research is therefore shifting from individual entities to whole systems, with the demand of extracting relationships between entities from biomedical text to generate knowledge. Biomedical Causal Relation Extraction (BCRE) aims to efficiently reveal high-quality relations from domain-related resources [28]. Transformer models, introduced in 2017 [29], have been developed to address NLP tasks and overcome the limitations of Recurrent Neural Networks (RNNs). Transformers have become the deep learning model of choice for NLP problems [30], [31] (Fig. 2).

Transformers make use of multi-headed self-attention to perform sequence-to-sequence learning tasks [29]. Self-attention is used to learn long-range dependencies between the elements in a sequence. Multi-head self-attention is the combination of several attention heads. The attention mechanism performs a lookup producing a set of weights for each element. The most relevant elements have the highest attention scores. This allows the model to be explainable with reference to both input and output. This has led to the development of pre-trained systems such as the popular BERT (Bidirectional Encoder Representations from Transformers) [32] and GPT (Generative Pre-trained Transformer) [33].

Researchers have been actively advancing BERT-based models to effectively and efficiently process textual information about biomedical and clinical texts. Notable examples of successful BERT-based models in this domain are:

• **SciBERT**[34] is an extension of the foundational BERT architecture, tailored to enhance its performance when dealing with scientific data.
• **BioBERT**[35] is a BERT model customized for the biomedical domain. It has been pre-trained on a substantial volume of biomedical text corpora, making it well-suited for biomedical text analysis.
• **ClinicalBERT**[36] serves as a language representation model designed specifically for extracting relationships between medical concepts from clinical notes.

Recent studies [37] show that these specifically pre-trained models outperform classical models in extracting evidence from biomedical corpora. The literature review shows that the application of NLP and DNNs on medical devices (especially software) is still in the embryonic phase.

## 3 Materials and methods

### 3.1 Proposed framework

**Figure 3:**
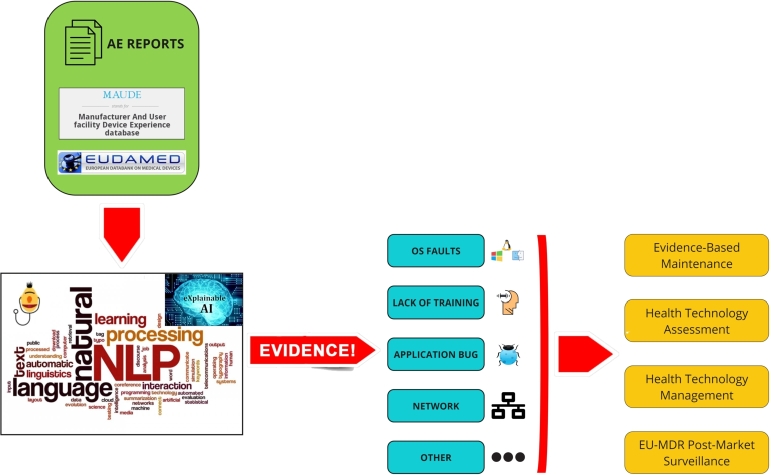
Proposed framework.

The proposed framework is illustrated in Fig. 3. Input data consist of medical device adverse event reports extracted from the US MAUDE database.

The developed model is based on the emilyalsentzer/Bio_ClinicalBERT model from HuggingFace, initialized from BioBERT (BioBERT-Base v1.0 + PubMed 200 K + PMC 270 K) and trained on all notes from MIMIC III, a database containing EHRs from Intensive-Care Unit patients at the Beth Israel Hospital in Boston, MA [38]. The model uses 12 layers of transformers block with a hidden size of 768 and a number of self-attention heads as 12. The pre-trained Bio_ClinicalBERT model has been fine-tuned on 3,705 manually-labelled adverse events reports extracted from the MAUDE database, to help the model learning domain-specific knowledge and terminology, leading to more accurate predictions. The implemented model performs binary text classification between HIT and non-HIT adverse event reports. XAI is also applied to the model to understand the weights of each feature related to the output classes. Weighted keywords extracted from both output classes may also be used to help users labelling new records, providing a prediction classification score. The developed framework represents a novelty in the Clinical Engineering field, as the RWE extracted can be applied for EBM, HTM, HTA, and PMS scopes as mentioned in Section 1.

### 3.2 Dataset statistical analysis

**Figure 4:**
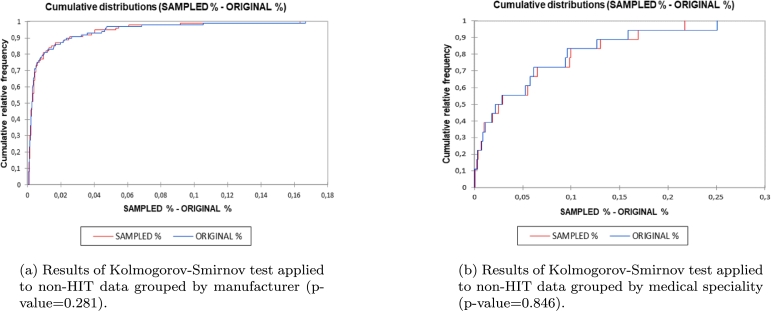
Results of Kolmogorov-Smirnov test applied to non-HIT data.

1,857 reports extracted from the FDA MAUDE were labelled by experts as HIT-related between January, 1st 2008 and June, 30th 2010. The remaining 513,183 reports which belonged to the same time span were otherwise classified as non-HIT. After discarding the records with no narrative data associated, the resulting dataset contained 492,030 records. 1,848 reports have been randomly sampled from the non-HIT population in order to build a balanced training dataset for the BERT classifier (class weights are respectively 1.0132 and 0.9871 for HIT and non-HIT adverse event reports). Kolmogorov-Smirnov test has been performed to compute the distances between the empirical (sample) and the theoretical (original) distributions and check whether the two follow the same distribution in relation to the manufacturer and the medical speciality (Fig. 4).

**Figure 5:**
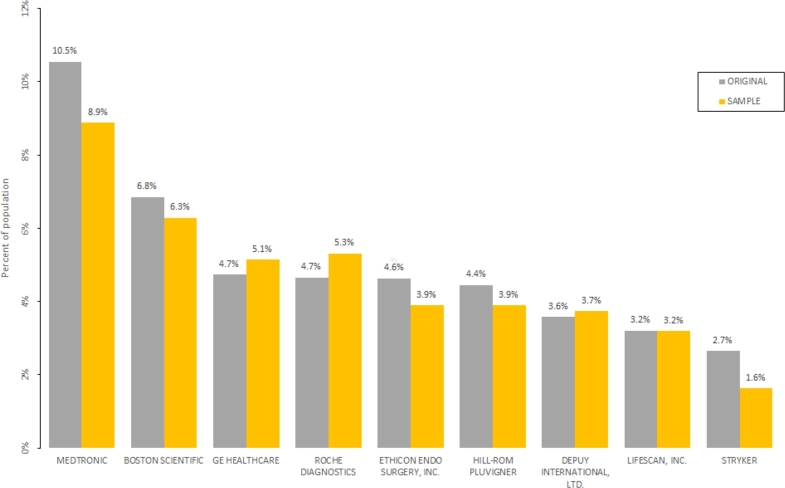
Bar chart of the percentage of original and sampled datasets for the top-10 manufacturer classes.

**Figure 6:**
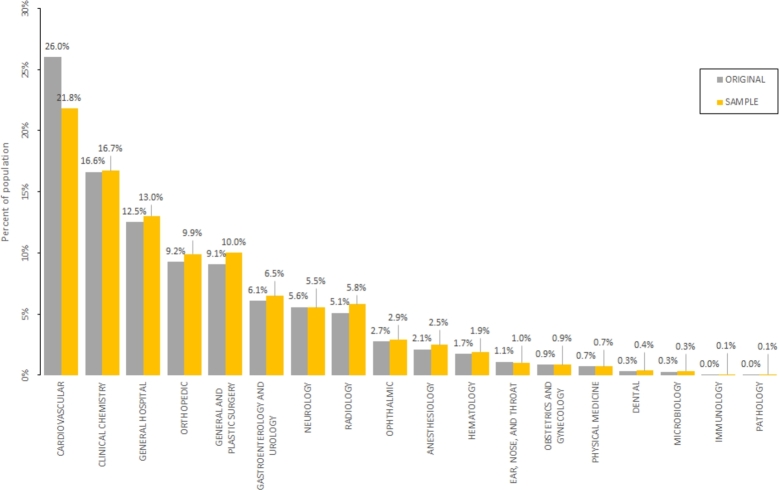
Bar chart of the percentage of original and sampled datasets for the identified medical specialities.

Manufacturer and medical speciality categorical variables were statistically described as frequency count and percentage (Figure 5, Figure 6).

**Figure 7:**
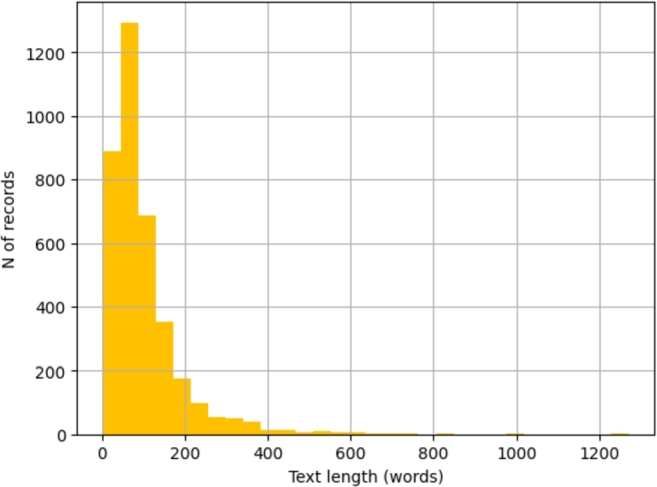
Number of words for analysed records. The majority of records (99.14%) present a text length which is shorter than 512 words.

Texts longer than 512 words have been truncated without losing any meaningful information as they represent 99.14% of the whole dataset (Fig. 7).

The combined reports have been used for training, evaluation, and testing on an NVIDIA GeForce RTX 3090. The model has been tested on 741 never seen records (20% of the whole dataset). The remaining samples (2,964 rows) have been split respectively into 80% for training and 20% for validation.

### 3.3 Explainable artificial intelligence

Two surrogate XAI models have been used to understand which are the main features that affect the output of the model, in order to unravel the decision-making process: LIME and SHAP.

**LIME** stands for Local Interpretable Model Agnostic Explanation. The “local” aspect means that it is used to explain individual predictions of a machine learning model. Each text record within the test set is explained in terms of keywords, each one weighted in terms of relevance to the contribution to the final binary classification [39].

**SHAP** (SHapley Additive exPlanations) is a method based on cooperative game theory and used to increase the transparency and interpretability of machine learning models. The absolute SHAP value shows how much a single feature affected the prediction [40].

## 4 Results

**Figure 8:**
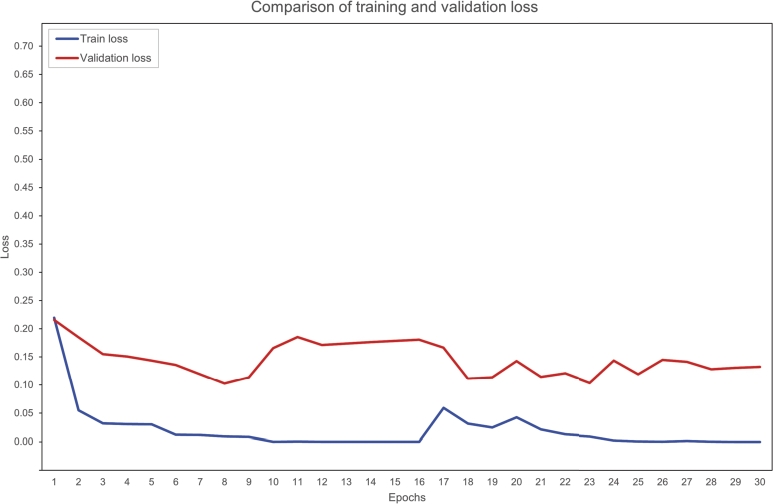
Comparison of training and validation loss during 30 epochs of training.

Various experiments have been initially conducted in order to tweak the model parameters. Dropout hyper-parameters have been constantly set to 0.5 for the attention layer and 0.1 for the hidden layer [41]. Three different activation functions - the Sigmoid Linear Unit (SiLU), the Rectifier Linear Unit (ReLU), and the Gaussian Error Linear Unit (GELU), three learning rates for the optimisation algorithm (5e^-5^, 3e^-5^, and 2e^-5^), and three batch sizes (8, 16, and 32) have been tested as suggested by Devlin et al. [32]. Tests have been also conducted on the number of frozen layers to achieve the best performance: 0, 4, 8, or 12 encoder layers have been frozen. A test has been also conducted by only freezing the embedding layers. The best performances have been achieved with 8 frozen layers, the GELU activation function, a batch size of 16, and the AdamW optimizer with a learning rate of 2e^-5^. The model has been trained for a total of 30 epochs. Fig. 8 plots the comparison between training and validation loss, showing how the model begins to overfit after the first seven epochs.

The observed trend is coherent with the general approach of fine-tuning BERT-based models for just a limited number of epochs [32]. Therefore, the model has been trained only for seven epochs to avoid overfitting, obtaining 0.9680 accuracy, 0.9603 precision, 0.9764 recall, and 0.9683 F1 score. Finally, a new model has been trained on the same dataset with the same tuned hyper-parameters and k-fold cross-validation (10 folds). Results are shown in Table 1.

**Table 1:** Results of 10-fold validation on 2,964 records. Highlighted fold 4 shows the best overall metrics.

Fold	Train loss	Validation loss	Accuracy	Precision	Recall	F1 score
1	0.0385	0.1277	0.9663	0.9860	0.9463	0.9658
2	0.0145	0.0169	0.9966	1.0000	0.9933	0.9966
3	0.0026	0.0102	1.0000	1.0000	1.0000	1.0000
**4**	**0.0001**	**0.0012**	**1.0000**	**1.0000**	**1.0000**	**1.0000**
5	0.0002	0.0088	1.0000	1.0000	1.0000	1.0000
6	0.0001	0.0028	1.0000	1.0000	1.0000	1.0000
7	0.0002	0.0026	1.0000	1.0000	1.0000	1.0000
8	0.0037	0.0047	0.9966	0.9933	1.0000	0.9966
9	0.0002	0.0143	1.0000	1.0000	1.0000	1.0000
10	0.0001	0.0014	1.0000	1.0000	1.0000	1.0000

### 4.1 Testing and performance evaluation

**Figure 9:**
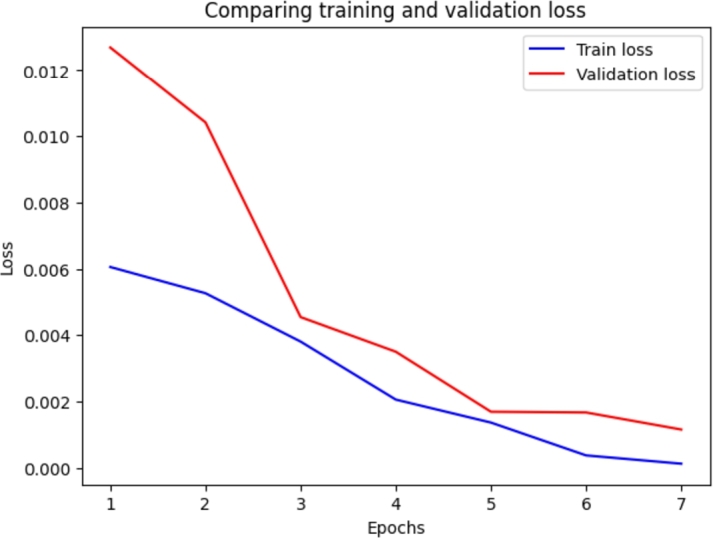
Comparison of training and validation loss for fold 4. Both losses decrease during the epochs so that it can be asserted that there is no overfitting.

**Figure 10:**
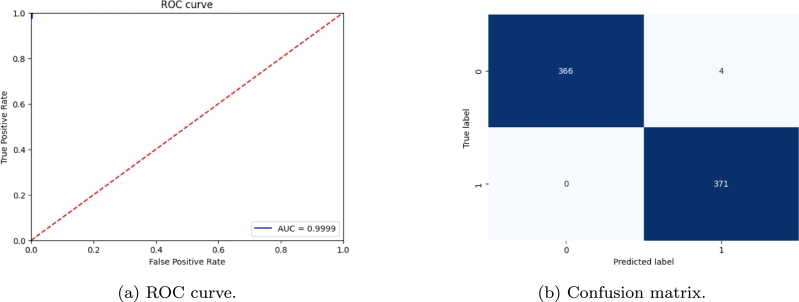
ROC curve and confusion matrix for fold 4 tested on 741 records (20% of the whole dataset).

The best-performing model (fold number 4) has then been tested on the testing dataset, with the following performances: 0.9946 accuracy, 0.9893 precision, 1.0000 recall, 0.9946 F1-score, and 98.93% Matthews Correlation Coefficient. Fig. 9 compares the training and validation loss in relation to the training epochs to ensure that there is no overfitting during training. Receiver Operating Characteristic (ROC) curve and confusion matrix for testing results are shown in Fig. 10.

The developed model has an overall classification run-time of 9.73 s ± 21.5 ms for 1,000 reports. The classification run-time of one report is 9.48 ms ± 5.6 μs. Results show better metrics than other existing HIT adverse events reports text classifiers based on non-BERT NLP models (Table 2).

**Table 2:** Comparison of performances of the proposed NLP model (fine-tuned ClinicalBERT) and other non-BERT models. LR - Logistic Regression. SVM - Support Vector Machine. CNN - Convolutional Neural Network. HRNN - Hierarchical Recurrent Neural Network.

Model	Accuracy	Precision	Recall	F1 score
**ClinicalBERT**	**0.9946**	**0.9893**	**1.0000**	**0.9946**
LR [42]	-	0.9670	0.9420	0.9540
LR [43]	-	0.6940	0.8040	0.7450
SVM+LR+CNN [44]	0.9012	0.8796	0.8606	0.8700
LR+CNN+HRNN [12]	0.9030	-	-	0.8760

### 4.2 Explainable AI applied to the model

**Figure 11:**
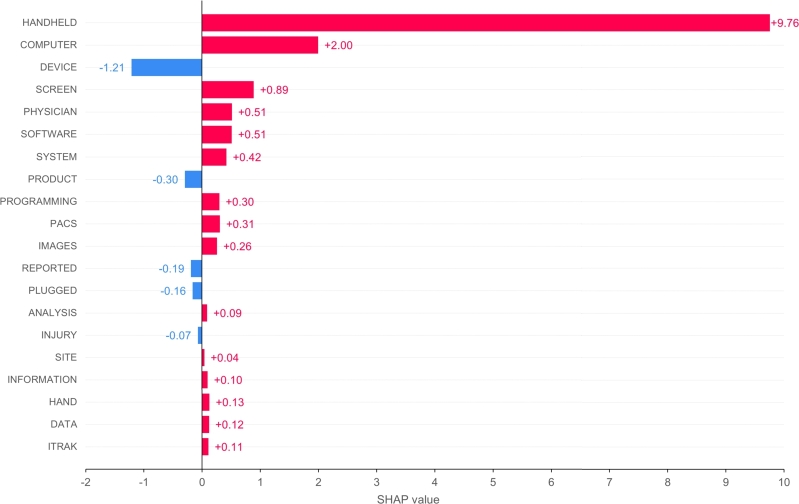
Bar plot of the top 20 features analysed with SHAP.

Fig. 11 shows the bar plot of the top 20 features obtained with SHAP applied on the best-performing model (fold 4) on the test set. Words such as “handheld”, “computer”, “screen”, and “software” have a high positive contribution to the prediction of the HIT class, while “device”, “product”, and “reported” have a negative contribution to the prediction, reflecting a positive weight for the non-HIT class.

**Figure 12:**
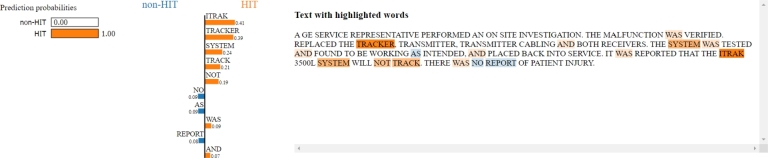
LIME applied to record with MDR key number 978,358 for top 10 features. Words like “track”, “tracker”, and “system” have a strong significance for the HIT output class, and they are those responsible for the final model classification.

Fig. 12 shows how LIME explains the output classification for a given text with the top 10 features: words such as “track”, “tracker”, or “system” have a high weight related to the HIT output class, so they are mainly responsible for the final classification of the model (which in this case is concordant with the label).

The overall weight for each keyword is calculated according to the formula:

$$F_{i}=\frac{\sum_{n=1}^{k}m_{n}}{k}$$

**Figure 13:**
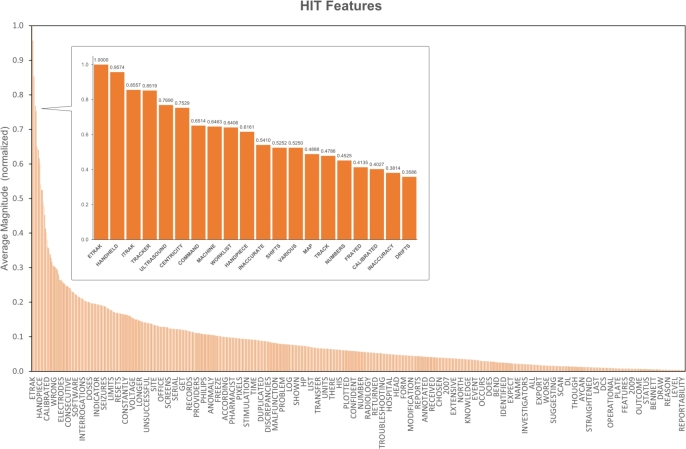
Bar plot for the keywords (features) related to HIT classification in relation to the average normalized weight, extracted with LIME. Top 10 features are highlighted in the callout.

**Figure 14:**
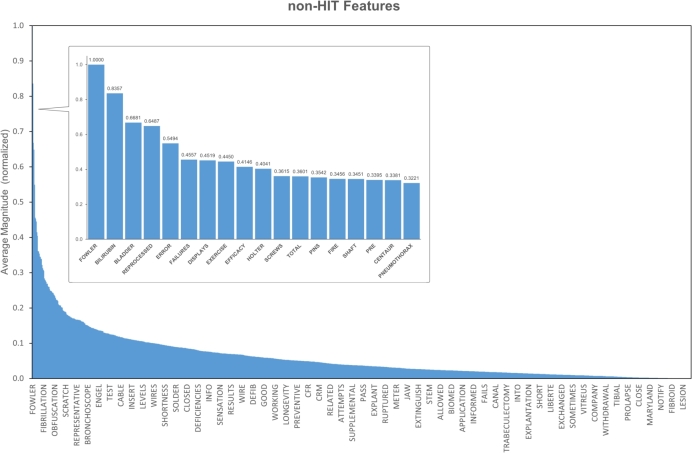
Bar plot for the keywords (features) related to non-HIT classification in relation to the average normalized weight, extracted with LIME. Top 10 features are highlighted in the callout.

For each keyword, all the magnitudes identified within the texts $m_{n}$ are summed together and then divided for the total count of the analysed keyword *k*. The calculated value $F_{i}$ is the overall weight associated with the given keyword, representing the overall importance of that feature within the whole testing set in relation to the final classification. The process is applied both to HIT and non-HIT keywords (Fig. 13 and Fig. 14).

Words like “handheld”, “itrak”, “ultrasound”, or “centricity” are associated with high average magnitudes in relation to the HIT class. Words like “fowler”, “bilirubin”, “bladder”, or “reprocessed” have instead higher average magnitudes for the non-HIT class.

## 5 Discussion

The developed and tested classification model is able to identify HIT-related adverse events using free-text narratives while highlighting the main features (keywords) of the final score by using XAI methods, helping understand the type of the described adverse event. By focusing only on the free-text descriptions, the model can be applied to different adverse events reporting systems and databases making the model generalizable. Moreover, the classification run-time of 9.73 s ± 21.5 ms for 1,000 reports does not preclude the usability of the model, as the run-time is coherent with the amount of analysed data and the used GPU. The implemented model can be easily saved and exported for being used by different stakeholders for the purposes described in Section 1. The model can be consumed in the same framework through the pipeline object provided by HuggingFace's transformers library, which allows abstracting most of the complex code, offering a simple API for the text classification task. Moreover, the model can also be exported in the Open Neural Network Exchange (ONNX) file format, which allows consuming the model in a different framework, such as Microsoft ML.NET, improving usability and interoperability. However, the proposed model is still a prototype, so a usability evaluation should be conducted in its production stage to actually evaluate how it performs in more stressful conditions.

### 5.1 Implementation and best practice

The definition of the hyperparameters of the model followed the common best practice for fine-tuning BERT models as described by Devlin et al. [32]. All the tested values for the activation function, the batch size, the optimizer, and the learning rate are those suggested by the authors of the language representation model. Another common best practice is to use only a few epochs for fine-tuning BERT models for domain-specific tasks, as a pre-trained model usually requires a much smaller number of epochs than models trained from scratch. In fact, the authors of BERT recommend between two and four epochs [32]. Further training often translates to overfitting the data and forgetting the pre-trained weights (catastrophic forgetting), as our initial fine-tuning also shows (Fig. 8). A high variability in accuracy between runs with the same settings has also been observed. This instability is known since the release of BERT. While catastrophic forgetting and the small sizes of the dataset were first suspected as the causes of this instability, more recent work [45] suggests that optimisation difficulties leading to vanishing gradients are the actual reasons. This represented a huge issue during the development of the model, even though defining fixed seeds and implementing layer-freezing during the fine-tuning phase seemed to partially mitigate the problem.

### 5.2 Explainable AI

**Figure 15:**
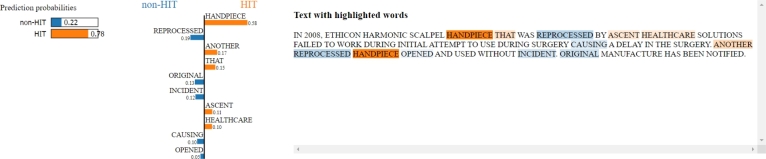
LIME applied to one adverse event report classified as HIT but labelled as non-HIT by experts. The misclassification is mainly due to the word “handpiece”.

XAI can help understand the process behind the final classification performed by the model. The two implemented strategies (SHAP and LIME) bring similar results for the most relevant features which led the model to the HIT class, while the results for the non-HIT class are different. The discrepancy may be mainly due to the different types of variables analysed in the charts. A single feature may have a not-high cumulative SHAP value (reflected in a not-high general weight) but with a high average magnitude due to its low frequency in the whole analysed corpora. It is also relevant to analyse what brought the model to perform an incorrect classification. We discuss the extracted texts analysed with LIME related to the four false positives classification (Fig. 10). In the first case, it clearly emerges that the word “handpiece” is the main responsible for the misclassification, because it has been commonly associated with HIT adverse events by the model during the training phase (Fig. 15).

**Figure 16:**
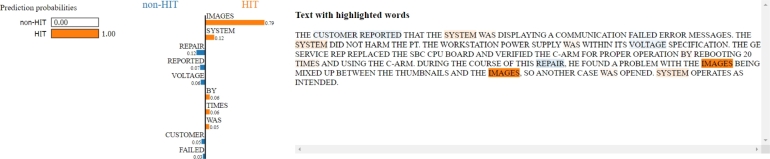
LIME applied to one adverse event report classified as HIT but labelled as non-HIT by experts. The words “images” and “system” strongly contribute to classifying the record as HIT adverse event. By reading the text, it can be assumed that the report is about a communication error with the subsequent mix-up of images, which can be actually considered a HIT-related adverse event.

**Figure 17:**
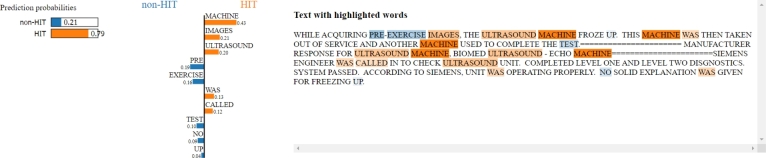
LIME applied to one adverse event report classified as HIT but labelled as non-HIT by experts. Words such as “machine”, “images”, and “ultrasound” lead the model toward a HIT prediction, which is actually true, being the initial label wrongly assigned as it emerges by reading the text.

The second and the third reports (Fig. 16 and Fig. 17) are totally different cases. In fact, for these reports, the initial label assigned by the experts is actually wrong, and the texts are correctly associated with HIT adverse events: a mix-up of images due to a communication error in the first example, and a system freeze issue during pre-exercise image acquisition in the second one.

**Figure 18:**
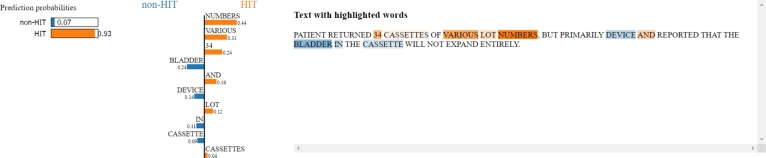
LIME applied to a false-positive classification. Features with higher weights such as “numbers”, “34”, and “various” lead the model toward the wrong classification, being the report related to a medication cassette reservoir.

In the last example (Fig. 18), it can be observed that the features with higher weights such as “numbers”, “34”, and “various” lead the model toward the wrong classification, being the report related to a medication cassette reservoir. Additional fine-tuning of the model should be performed in order to make it able to contextualize the words “numbers” and “various” more specifically, resulting in lesser associated classification weights as they are more common-speech features.

### 5.3 Environmental impact of artificial intelligence

As a final consideration, the environmental impact of AI should also be cited. Indeed, whenever AI models are described and analysed, a parallel discussion on the environmental impact of such technologies should be performed. CO_2_ emission from the world computing infrastructure is now equivalent to aeronautics at its top, and it is growing faster and faster each year [46]. According to Belkhir and Elmeligi [47] the demand for electricity from data centres will contribute to 14% of the global emission of greenhouse gas by 2040. Even only the training of a small NLP model can produce about 300,000 kilograms of CO_2_, which is the same as five gas-fuelled cars in their whole life cycle, or 125 flights from New York to Beijing and back [48]. In such a scenario, especially when discussing the scalability of an AI model to larger case studies and for bigger stakeholders, the authors think that the common practice of maximizing computing cycles to improve performances must not be the only goal, but it has to be combined with the analysis of the consumption of energy of CPUs and GPUs, not only from the economic point of view but also as a contribution to the global pollution.

## 6 Conclusion

The proposed framework employs NLP techniques and models, such as BERT, to automatically identify adverse event reports related to Health Information Technologies. Input data come from the FDA MAUDE database of medical device adverse event reports, but they can also originate from different sources. Further connections could be soon available to access data from local hospitals' CMMS and Computer-Aided Facility Management (CAFM) systems [49], [50], [51], [52]. The framework aims to extract RWE to support EBM of medical devices, HTA, and HTM, as well as PMS as outlined in the EU-MDR. The designed model uses a pre-trained version of ClinicalBERT, additionally fine-tuned on 2,964 adverse events reports extracted from the FDA MAUDE database, which had been previously manually labelled by experts. The model has then been tested with 741 reports. Results show better metrics than other existing NLP HIT adverse events reports text classifiers based on non-BERT models [12], [42], [43], [44]. Explainable Artificial Intelligence techniques have also been employed to understand how the model interprets each feature, calculating the overall weight of each word in relation to the final output classes. Both employed XAI methods (LIME and SHAP) highlight how a subset of specific features (e.g., “handheld”, “computer”, “software”) have a high weight in determining the final output class of the model, as it is conceivable and congruent with the type of analysed events.

Highlighting both the most common faults and the unexpected new challenges before introducing a new device is vital to perform an actual assessment of the whole life-cycle of the technology (from purchase to maintenance until discontinuation), evaluating all the possible hidden costs which it may impact. The performance and the robustness of the model can be further improved by exploiting new adverse event reports extracted by the MAUDE or other SRS databases (e.g., the EU EUDAMED and the Australian DAEN). In doing so, the results that emerged from the implementation of XAI methods can be incorporated to ease the process of labelling new records.

## CRediT authorship contribution statement

**Alessio Luschi:** Conceptualization, Formal analysis, Investigation, Methodology, Software, Writing – original draft, Writing – review & editing, Data curation, Resources. **Paolo Nesi:** Investigation, Methodology, Supervision, Validation. **Ernesto Iadanza:** Conceptualization, Methodology, Project administration, Supervision, Validation.

## Declaration of Competing Interest

The authors declare that they have no known competing financial interests or personal relationships that could have appeared to influence the work reported in this paper.

## Data Availability

The data that support the findings of this study are available in GitHub at DOI/10.5281/zenodo.10041106. Data includes the labelled dataset extracted from the MAUDE database, the implemented model, and the Python code.

## Appendix A Supplementary material

Labelled dataset Labelled dataset extracted from the FDA MAUDE database. Labelled dataset

Jupyter Notebook Jupyter Notebook with Python code. Jupyter Notebook
